# AEDNav: indoor navigation for locating automated external defibrillator

**DOI:** 10.1186/s12911-022-01886-7

**Published:** 2022-06-20

**Authors:** Gaurav Rao, Vijay Mago, Pawan Lingras, David W. Savage

**Affiliations:** 1 Department of Mathematics and Computing Science, Saint Mary’s University, Halifax, NS Canada; 2 Department of Computer Science, Lakehead University, Thunder Bay, ON Canada; 3 Northern Ontario School of Medicine, Thunder Bay, ON Canada

**Keywords:** Automated external defibrillator (AED), Sudden cardiac arrest (SCA), Indoor navigation, Wi-Fi, Magnetic data, Dynamic time warping (DTW), Wi-Fi fingerprinting

## Abstract

**Background:**

In a sudden cardiac arrest, starting CPR and applying an AED immediately are the two highest resuscitation priorities. Many existing mobile applications have been developed to assist users in locating a nearby AED. However, these applications do not provide indoor navigation to the AED location. The time required to locate an AED inside a building due to a lack of indoor navigation systems will reduce the patient’s chance of survival. The existing indoor navigation solutions either require special hardware, a large dataset or a significant amount of initial work. These requirements make these systems not viable for implementation on a large-scale.

**Methods:**

The proposed system collects Wi-Fi information from the existing devices and the path’s magnetic information using a smartphone to guide the user from a starting point to an AED. The information collected is processed using four techniques: turn detection method, Magnetic data pattern matching method, Wi-Fi fingerprinting method and Closest Wi-Fi location method to estimate user location. The user location estimations from all four techniques are further processed to determine the user’s location on the path, which is then used to guide the user to the AED location.

**Results:**

The four techniques used in the proposed system *Turn detection*, *Magnetic data pattern matching*, *Closest Wi-Fi location* and *Wi-Fi fingerprinting* can individually achieve the accuracy of 80% with the error distance ± 9.4 m, ± 2.4 m, ± 4.6 m, and ± 4.6 m respectively. These four techniques, applied individually, may not always provide stable results. Combining these techniques results in a robust system with an overall accuracy of 80% with an error distance of ± 2.74 m. In comparison, the proposed system’s accuracy is higher than the existing systems that use Wi-Fi and magnetic data.

**Conclusion:**

This research proposes a novel approach that requires no special hardware, large scale data or significant initial work to provide indoor navigation. The proposed system *AEDNav* can achieve an accuracy similar to the existing indoor navigation systems. Implementing this indoor navigation system could reduce the time to locate an AED and ultimately increase patient survival during sudden cardiac arrest.

## Background

### Sudden cardiac arrest

**Fig. 1:**
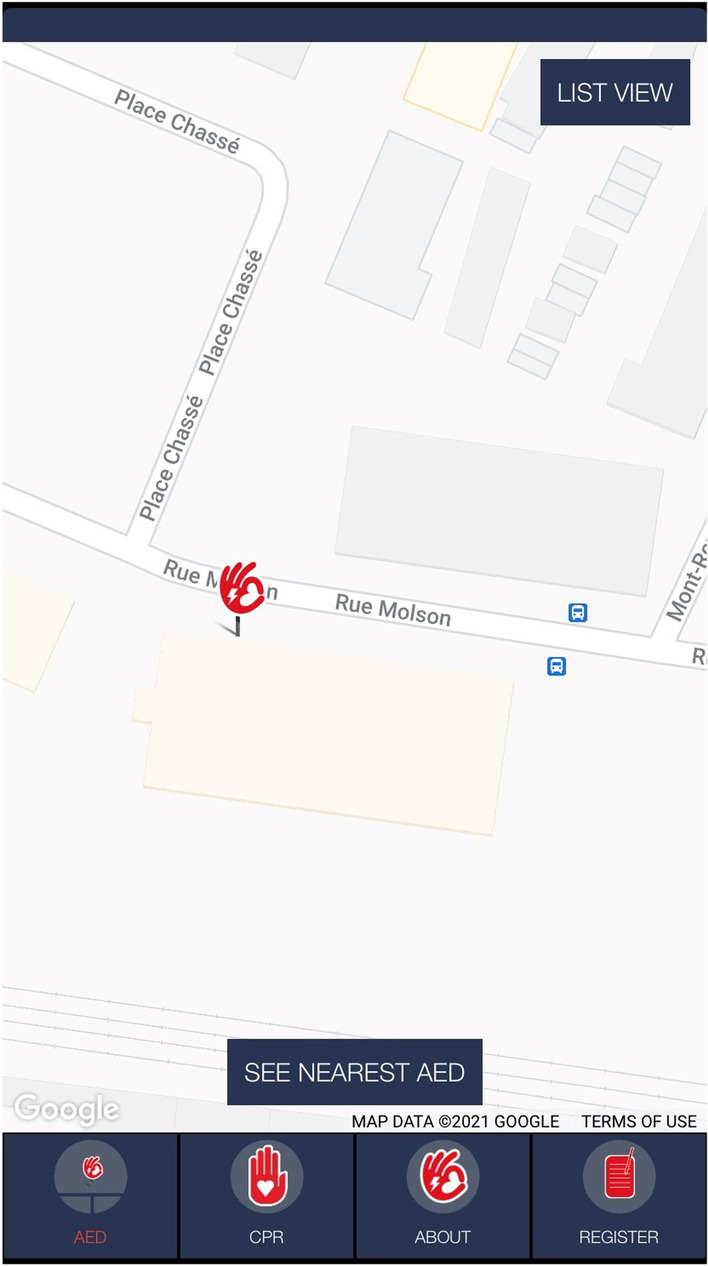
Screenshot from AED Quebec Mobile application. The screenshot displays an AED location in an 3271 m^2^ building

In sudden cardiac arrest (SCA), a patient’s heart beats in an irregular rhythm or stops beating [1]. In either case, the patient should be immediately treated with cardiopulmonary resuscitation (CPR) and with an automated external defibrillator (AED) if clinically indicated [2, 3]. More than 350,000 deaths in the U.S. occur due to SCA and more than 40,000 in Canada annually [4–7]. According to the American Heart Association, the chances of surviving an SCA decrease by 10% per minute [2, 3, 8–10], and studies also confirm that the patient’s survival increase by up to 24% if a bystander provides CPR before the arrival of emergency services [3, 11, 12]. To reduce the response time, some emergency services have implemented Responder Network Systems (RNS) [12–15]. The emergency call receivers activate the RNS systems. The RNS system then alerts all the registered users in the patient’s vicinity to assist the patient either by providing CPR or transporting an AED to the patient. For locating nearby AEDs, there are several mobile applications such as AED Quebec, Staying Alive, and Pulse Point Respond [16–18], that displays nearby AEDs on a map. Using these mobile applications, the user can locate the nearest AED and navigate to them using mapping applications such as Google Maps (Android/iOS) [19], Apple Maps (iOS) [20], and Waze (iOS) [21]. These applications can guide the users to the mapped address only, however, users then have to locate the AED within the building. Previous research has shown that only 16% of the AEDs are located within a visible area (e.g., building entrance, front desk, or lobby) [22]. For the remaining 84% of AEDs in the community, the user will require time to locate the AED inside the building. Figure 1 shows a screenshot from the AED Quebec [16] mobile application displaying an AED location. The building referred is approximately 3271 m^2^ in size and has two floors. Finding the AED in this building will be a challenging and time-consuming task. In such cases, indoor navigation can help the user locate the AED quickly and ultimately reduce the time to start the patient’s treatment and therefore, increase their chances of survival.

#### Navigation and sudden cardiac arrest

Navigation has always been an essential part of daily life and is becoming difficult with the ever-increasing urban growth. For navigation, a system requires a map and the user’s location to the map for providing directions. A map of the location can be built upon its physical features such as longitude, latitude, and altitude information, measurement of the building (meters, feet, yards), magnetic information inside a building, and Wi-Fi information inside a building [23–25]. Navigation can be split into two categories: (1) outdoor and (2) indoor. Outdoor navigation systems can be more precise, supporting activities such as autonomous driving [26, 27]. These systems use the Global Positioning System (GPS) satellite network to localize the user’s position. Mobile applications such as Google Maps (Android/iOS) [19], Apple Maps (iOS) [20], and Waze (iOS) [21] have been using GPS to help users navigate while travelling. A significant drawback of this technology is that it requires a line of sight between the receiver (e.g., a smartphone, tablet, and smartwatch) and the satellites. Thus, it can be used only outdoors.

With large buildings such as malls, offices, and warehouses, indoor navigation has become an important research topic in recent years [28–30]. Researchers have proposed various ways to localize the user for indoor navigation. Chumkamo et al. used radio frequency identification devices (RFIDs) to localize the user’s location and the same technology to construct an indoor map of the building [31]. Huang et al. used Wi-Fi information to generate a map and to localize the user’s location [32].

During the literature review, indoor navigation systems were identified which can develop indoor maps for navigation for unmapped locations. The systems found in the literature have been grouped based on the technology they used. The following subsections explain the strengths of the existing systems, drawbacks, and a brief description of the proposed model.

#### Existing indoor navigation systems

RFID based indoor navigation systems use inexpensive and low-powered hardware devices that are installed at a particular location in the building [23, 31]. The system requires two types of devices: (1) readers—that need to be installed at fixed locations throughout the building and (2) RFID cards that the user will carry to communicate with the fixed readers. The RFID reader can read the card from a very short distance, thus making the system precise in locating the user’s position. However, these systems require significant setup, including having a floor plan of the building with each RFID reader’s location. Also, based on the physical size of the building, the hardware cost can increase substantially. This system will not be financially viable to implement on a large-scale (e.g., city-wide or countrywide).

An alternative solution to an RFID-based system was to use a Quick Response code (QR code) based system or a light-based system. The QR code system requires unique QR codes at visible locations on the pathway [33, 34]. The QR code system pre-requisite is to map the unique QR codes on the premises floor plan. The navigating user’s mobile device then scans the QR code, and the system determines the user’s location using the pre-built map. The light-based system requires replacing the overhead lights bulbs with a unique lighting tube that emits light similar to a barcode [35–37]. The barcode is not visible to the human eye, but it can be read using the smartphone camera and visual computing. This technology eliminates the need for special hardware requirements for the user. However, QR code-based systems are preferable to light-based systems as printing QR codes are cheaper than replacing light bulbs. In both systems, installation of the system is required, along with mapping the premises. The initial work will be very costly when done for an AED in a large building.

Although the existing indoor navigation systems described have been proven to work well, the large-scale setup of hardware required to deploy these systems across a city is cost-prohibitive. Artificial Intelligence (AI) driven camera-based indoor navigation systems are one potential system that does not require the extensive hardware setup of the other systems [38–42]. These systems require the first-time user to capture video of the path using their smartphone cameras. The recorded video is then processed to identify unique objects such as fire extinguishers, nameplates, exit signs and other prominent objects. These objects are then used to locate the user within the building as they walk along the path. However, this system has some drawbacks. If people or other objects obstruct the object identified by the system, then the user’s location will not be determined. The obstruction is a common situation in public buildings where people will be obstructing the line of sight between the object and the camera. The unique identifying objects, such as exit signs or fire extinguishers, can be at multiple locations in a building. Thus incorrect localization of the user is possible. Another issue is that the recording and processing of videos will be challenging for smartphones having low computing power, and the recording of videos may raise privacy concerns.

The Micro Electro Mechanical Systems (MEMS) and Wi-Fi-based technology are two potential solutions for the computation and privacy issues associated with camera-based systems. These technologies have been examined extensively. The MEMS is a micro-electrical and mechanical system constructed at a miniature scale. These systems are small and can be installed in small devices such as smartphones and smartwatches to capture the required data with very little power consumption. Most standard smartphones have the following MEMS sensors: accelerometer, gyroscope and magnetometer. These sensors can provide the device’s acceleration, orientation and magnetic field information. The accelerometer could be used for user localization, as speed can be calculated from the acceleration and distance. However, in practical use, the accelerometer generates significant noise, which cannot be removed from the signal and over short distances results in inaccurate data [43–45]. The noise in accelerometer data is primarily caused due to multiple factors: (a) miscalibration of the device, (b) white noise, and (c) vibration in handling the device. Some of this noise can be removed using digital signal processing but not all. Therefore, none of the navigation systems proposed in the literature use accelerometer data solely. However, some researchers use the acceleration information and the dead reckoning method to localize a user between two reference points that are closeby [43–45]. Another approach that uses accelerometer data is identifying the number of steps in the data and then calculating the distance [43, 46, 47]. An issue with this approach is that users do not always walk with the same stride length. The stride length differs between users, time of day, walking surface, walking speed and many other factors. Thus, there is uncertainty in how accurate this system will be in measuring distance.

Researchers have also proposed indoor navigation using magnetic field information [24, 47, 48]. These systems require collecting magnetic information along a path and the user using it to navigate. Imran et al. proposed a system called mPILOT, this system used machine learning algorithms to localize the user based on magnetic information [49]. Chi et al. proposed a GROPING system; this system used a crowdsourcing technique to collect magnetic information [50]. The systems mentioned above and other systems proposed in the literature require a large amount of data for training the system. This requirement is a significant drawback when these systems need to be implemented in a large physical area. Wi-Fi-based indoor navigation systems are another potential method for indoor navigation [22, 51–53]. These systems use Received Signal Strength Indicator (RSSI) values for the access points and create a reference point in the path. RSSI values represent the strength of a particular signal. However, this system cannot be used alone to provide full navigation, as the Wi-Fi scan takes several seconds to scan and collect RSSI values of all available access points. In theory, if additional information, such as the transmission power, receiver power of the devices, obstructions in the pathways, and other environmental details, are available, then the distance between the access point and the receiver can be found. In real-life situations, the environment is disturbed by nearby users, airflow, refraction and reflection of signals. Thus, RSSI values can be considered a reference point but not suitable for measuring distances. To build a continuous indoor navigation system, researchers have proposed hybrid systems that use a combination of Wi-Fi, magnetic, and MEMS sensors [54–56]. In all of these systems, the system requires a large quantity of data to create the premises map, which is later used to provide navigation. This initial data requirement would be a significant challenge for indoor navigation systems when thousands of buildings need to be mapped.

#### Why are existing indoor navigation systems not suitable for navigating to AEDs?

AEDs are generally installed either by the owner or a contractor. The AED can be placed at a fixed location (e.g., on a wall) or a temporary location (e.g., cabinet). In both cases, it requires a person to install it, and this person is the first person to know the location. After installation, the AED will be accessed only at the time of its maintenance (i.e., six months to two years for battery or pad replacement) or during an emergency. Therefore, the opportunity to collect data for the navigation path is possible only at the installation time. Since one or two individuals do the AED installation, they can potentially collect only a handful of datasets for the entire path.

The requirements for the proposed system are:

- No hardware dependency on the premises
- No special hardware required for the end-user
- Minimum initial work for collecting the path
- Scalable and easy implementation
- Minimum maintenance

**Table 1:** A comparison showing existing indoor navigation technologies

	RFID	QR code	Camera	MEMs	Wi-Fi
Premises hardware required	Yes	Yes	No	No	Yes
Cost of hardware	High	Low	None	None	Use existing
Initial data	Small	Small	Medium	Large	Large
Require floor plan	Yes	Yes	No	No	No
Computation	Low	High	High	Low	Low
Initial work cost	High	High	Low	Low	Low
Privacy issues	No	No	Yes	No	No

Red text highlights drawbacks when implementing the technology to find an AED in malls/high-rising buildings or on a large scale such as city

Table 1, compares the different technologies that are used to provide indoor navigation. These are not compatible for navigating users to the AED, as one or more criteria are not viable for implementation. Based on the existing systems’ learnings, the Proposed Solution subsection provides the implementable solution in locating the AED.

#### Summary of the proposed solution

In this paper, we propose a system called *AEDNav* to fulfill requirements for an indoor navigation system to locate an AED. This system will use the magnetic field and Wi-Fi information of the premises collected by an installer through a smartphone. The magnetic field information will be used to generate a map, and the Wi-Fi information will be marked on the generated map as reference points. For a potential user seeking to find an AED, the user’s smartphone will collect the magnetic field and Wi-Fi information. The collected information will be transmitted to a server, and the server will then compare the information with the previously collected information. After the comparison, the system will determine the path covered by the user and provide the directions for navigation to the AED.

Achievements and novelty of the proposed system *AEDNav*:

- *AEDNav* requires only one dataset describing the path to provide navigation.
- The end-user will be able to navigate to the AED using their smartphone.
- *AEDNav* is highly scalable and cost-effective.

## Methods

### Overview

Based on the survey of existing indoor navigation technologies in the literature, the authors have chosen to use MEMs and Wi-Fi sensors to localize and navigate the user to the AED. The main reason for this choice was that these technologies are available in a standard smartphone, and the AED installer and subsequent users can use their smartphones to collect data and navigate to the AED, respectively. Most buildings have Wi-Fi access points available for public or private use. The proposed solution uses the RSSI information of the access point, which can be obtained from both private and public access points without establishing a data connection with them. The authors would like to emphasize that establishing a data connection is not required by the proposed system. However, the system can work without the RSSI information.

The magnetometer information is used to determine the patterns and directions of the path. The sequence of direction or elevation changes is generated based on the magnetic altitude, magnetic latitude, magnetic longitude, and magnetic direction information of the path. The magnetic information of a physical location varies depending on the physical environment, especially if a large electronic or magnetic device is nearby (e.g., a refrigerator, speakers, or air conditioning unit). Thus, the magnetic information can help map the premises with landmarks to estimate a user’s location. Like other MEMs sensors, magnetic sensors also have noise and high volatility in the sensor data. The *AEDNav* recognizes this issue and the mitigation strategy is discussed in the Data Cleaning subsection.

The Wi-Fi RSSI information is added to the proposed system for creating reference points along the path. A reference point is a marker on the collected magnetic data where the Wi-Fi information is received from the mobile device. The reference point created using this technique helps in narrowing the path to find a Wi-Fi device with an appropriate RSSI value. The reference points are essential because the magnetic information is a continuous stream of information and is used to determine the path’s patterns. However, there can be sections in the data where no pattern is identified. In such situations, reference points will provide a landmark to localize the user’s location along the path. For example, if a user is walking in a straight line with no magnetic disturbances, then the magnetic data will not locate the user on the path. However, if RSSI information is captured along those paths, the collection points can provide landmarks for the path travelled. Theoretically, the RSSI of a particular Wi-Fi should be at a fixed distance from the sender (access point) in any direction. Unfortunately, the user’s distance to a single access point cannot be calculated due to the user being in a 3-dimensional space. To use the RSSI to determine the user’s location, triangulation of multiple access points is needed. The RSSI values are interrupted by the environment, weather, and people in the building. Therefore, matching several RSSI values does not help determine the closest access point. In the proposed solution, the RSSI information is filtered to capture different metrics used to locate the user. The RSSI filtering and cleaning details are discussed in the *Data Cleaning* subsection.

#### Data collection

**Fig. 2:**
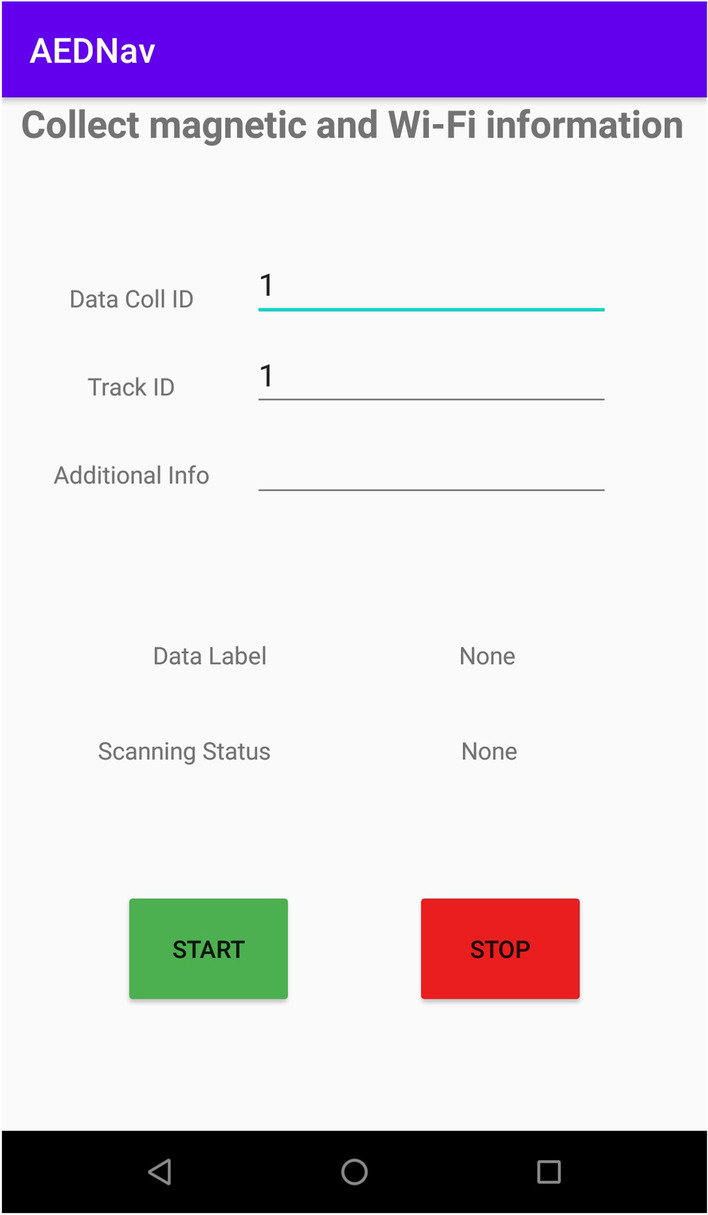
A screenshot from the Android app created to collect the magnetic and Wi-Fi data

**Fig. 3:**
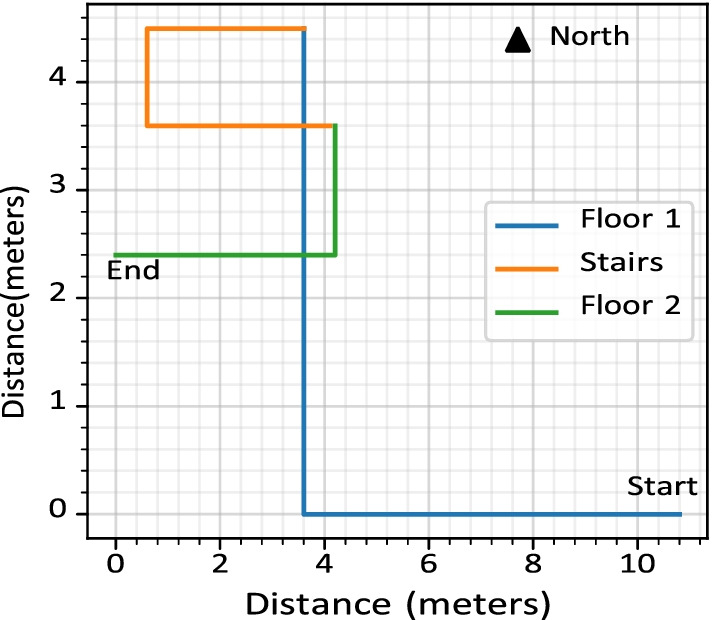
The path used in this study to collect data

**Fig. 4:**
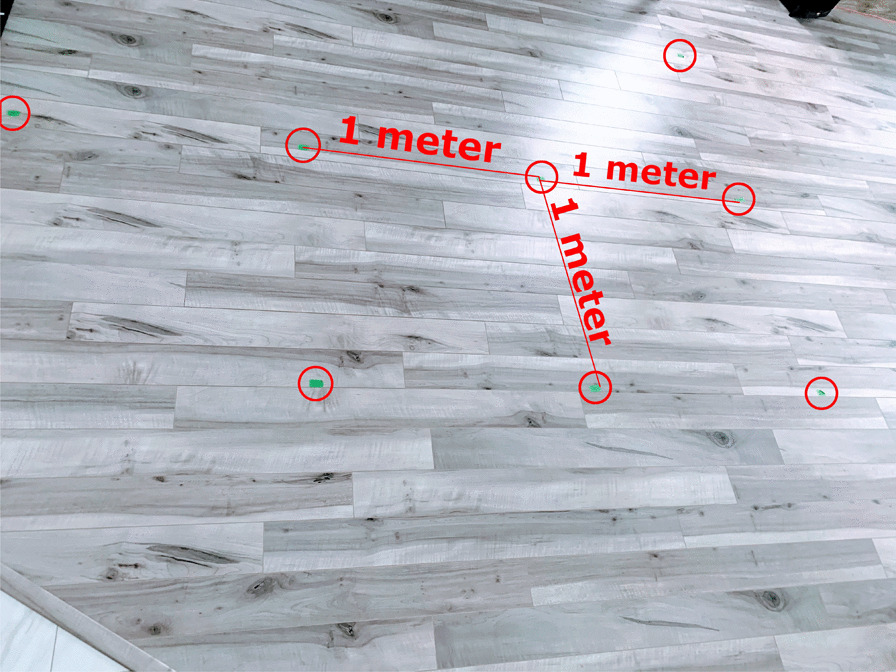
The walking path was mapped using green tape for the user’s to follow

During this study, an Android mobile application was developed to collect the data. Figure 2 is a screenshot of the Android application. Each user’s walk data recording is assigned a unique *Data Coll ID*. The *Track ID* is used to differentiate the different tracks travelled during the study. The *Additional Info* field was used to record additional information related to the track or user’s comments. The *Data Label* field is auto-populated upon the start of the data collection process, and it displays the unique label generated for the current data collection. The *START* and *STOP* buttons control the data collection process. All collected data is stored in a comma-delimited (i.e., CSV) file on the mobile device and later exported to a laptop for analysis.

The features captured for the Wi-Fi scanning include Service Set Identifier (SSID), Basic service set identifiers (BSSID), Received Signal Strength Indicator (RSSI) and the frequency band (2.4 GHz or 5 GHz) of each access point found during scanning. The features captured for the magnetic data collection include magnetic longitude, magnetic latitude, magnetic altitude, and magnetic field strength. The data mentioned above were collected using standard Android APIs.

This research was conducted during a time of significant restrictions due to Covid-19, therefore, the authors had access to limited physical locations and limited number of participants to test the system. For this study, the data was collected from a track inside a home. Figure 3 shows the path used to collect the data. The length of the path is 24.9 m. The path was marked using green tape such that each user’s walk is aligned, as shown in Fig. 4. Three adults living in the home participated in collecting the data. Each of the three participants walked the predetermined path 20 times and used the same Google Nexus 5X device with the application developed to collect the magnetic and RSSI data.

**Fig. 5:**
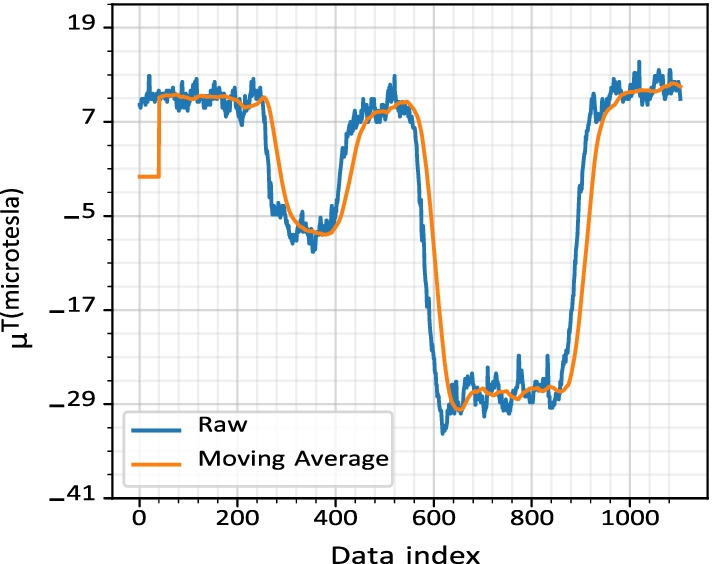
Moving average of the raw signal with a window size of 40

#### Data cleaning

The magnetic and RSSI data collected using the mobile device contains significant noise. The noise in the data is from multiple sources including: (1) calibration of the hardware, (2) vibration of the participant’s hand while holding the device, (3) environmental factors such as reflection and refraction of signals, (4) obstruction caused by objects or user’s movement between the sender (i.e., access point) and receiver (i.e., smartphone), (5) electrical or magnetic objects nearby, and (6) sensor initialization. Much of the noise can be reduced by calculating the signal’s moving average, shown in Fig. 5. The extreme noises in the signal are filtered out using rule-based methods. More information is provided below.

*Magnetic data cleaning* The extreme values in the magnetic data were filtered out using a rule-based method. During sensor initialization, magnetic values are set to zero. These values were filtered out using the following rule: exclude records having magnetic altitude, magnetic latitude, and magnetic longitude equal to zero. For small amounts of noise, the filtered signal is passed through a moving average function. The moving average window size is set to 40 because the data collection frequency for the magnetic data is 40hz. Within a second of an average walk, a large object can be detected, and smaller vibration peaks can be averaged out using the moving average filter.

1 $$\begin{aligned} {\text {FSPL (dB)}} = 10{\log }_{10} \Bigg ( {\bigg ( {\frac{4 \pi df}{\text {c}}} \bigg )}^2\Bigg ) \end{aligned}$$

*RSSI data cleaning* A rule-based filter was applied to the RSSI data for excluding records with an RSSI value larger than $-$30. The value $-$30 is selected based on the Free Space Path Loss (FSPL) of a signal. According to the FSPL Eq.  (1), if the RSSI value is $-$30, then in an average household environment, the distance between the access points and the receiving device is 0 m [57, 58]. Therefore, an RSSI value of $-$30 or higher is an extreme value and thus ignored. Further cleaning of the data was performed by checking the footprint of access points in multiple locations. In this step, the data of a particular access point is excluded if it is not available in 80% of the locations. This filtration helps remove access points that are far from the user and can cause a significant variation in the euclidean distance calculation. In the experimentation dataset, the filtration process excluded 73% of access points from a total of 72 access points.

#### System architecture

**Fig. 6:**
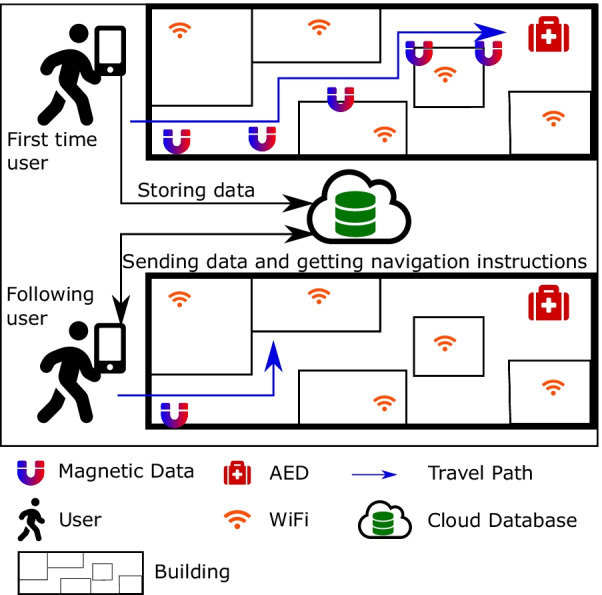
Workflow showing data collection and its usage

It is assumed that the AED installer collects the path’s initial data from outside the building to the AED. The installer can be a contractor or the owner of the AED. The installer will start collecting the data outside of the building to determine the installer’s initial location using the GPS. The GPS location is precise and thus will be used as the starting point of the track. After the GPS location is determined, the developed mobile application will start recording the magnetic and the Wi-Fi information until the installer arrives at the AED’s location. After completing the path, the collected information is transmitted to a remote server. When a user is searching for the AED, they will use the designed mobile application for indoor navigation. The mobile application first matches the user location with the initial GPS location of the track. Once the initial locations are matched, the mobile application will collect the magnetic and the Wi-Fi information and send it to the server for navigation directions. The server will compare the input data with the pre-recorded track information to provide the navigation directions. Since the navigation computation will be performed on the server, the system can handle multiple navigational requests simultaneously, and if required, additional servers can be added to handle a large number of requests. The system architecture is shown in Fig. 6.

**Fig. 7:**
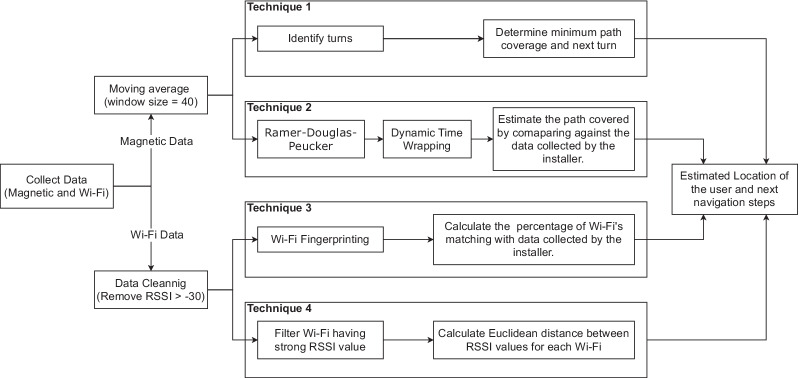
The system architecture to determine the path travelled by the user

#### Track comparison

The track comparison is performed between the pre-recorded track data and the data collected by the user who is following the path. The track comparison involves four different techniques to estimate the user’s location and provide them with the navigational steps. Figure 7 shows the four techniques and the steps involved in each technique. Two of these techniques use magnetic data, and the other two use Wi-Fi data to compute the estimated path travelled by the user finding the AED. None of the techniques used directly compare the raw values due to significant noise. Therefore, data patterns are identified based on the type of data collected and used for performing comparisons. Each of these techniques is explained in detail below.
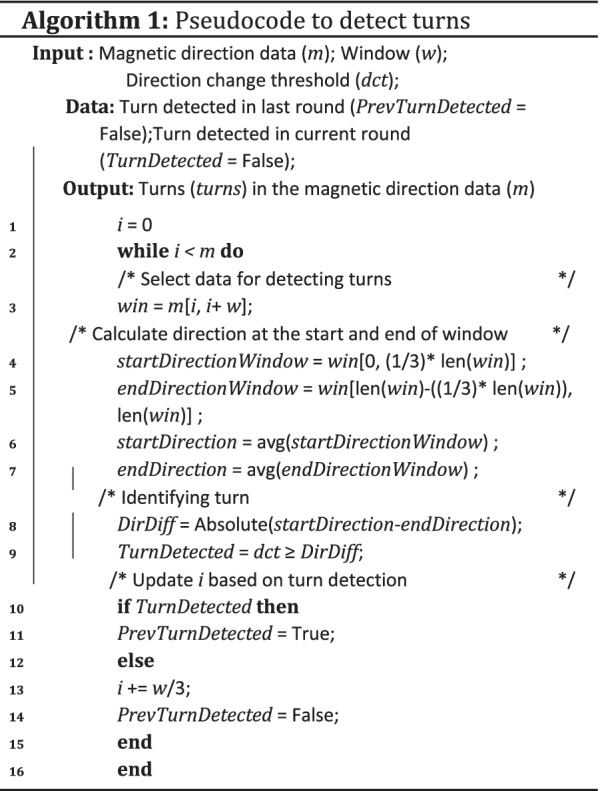

**Fig. 8:**
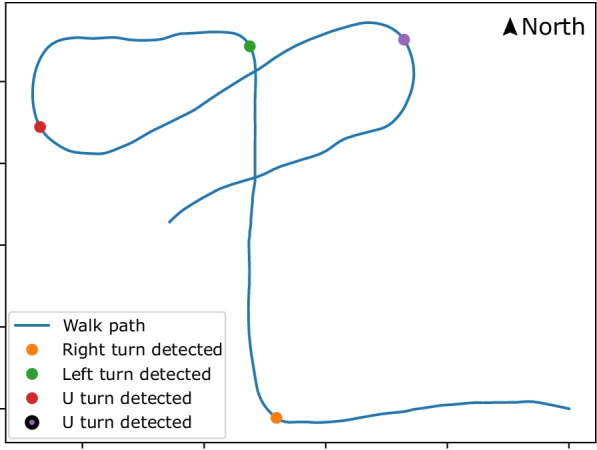
Map showing turns detected based on the magnetic information

1. *Turn detection and comparison* Turn detection is an essential part of the navigation process. Turns in a path can be used as reference points to determine the user’s position. Turns can also help in the dynamic time warping comparison of the signal ignoring the user’s walking speed and stop time during the walk. The magnetic direction data contains noise induced by the magnetic sensor and the vibrations caused by the user’s hand when standing or while walking. The peaks generated by the noise are removed by calculating the moving average of the data. The moving average window size is 40 due to the data frequency (i.e., 40 Hz) to reduce noise on turn detection. The authors propose an algorithm (Algorithm 1) to detect turns in the magnetic data collected. The turn algorithm loops through the data with a window of 2 s to detect a change in direction, line 2 in Algorithm 1. In the loop, the window data is divided into three equal parts, the difference between the average of the first and third parts of the data is used to determine a turn, line 4 and 5 in Algorithm 1. A difference of 30$^{\circ }$ is required for the algorithm to determine whether a turn was made, line 9 in Algorithm 1 . The minimum threshold of 30$^{\circ }$ will disregard noise in the data (e.g., small deviations in direction change when walking around an object) when walking a straight line. If a turn is detected, then the next window’s starting point is the previous window’s start. The window extension is performed to detect a *U-turn* (i.e., a 180$^{\circ }$ turn) in the data. A U-turn might take a few seconds to occur, and the 2-s window might not be sufficient to detect it. Figure 8, shows the turn detected in a path. During the comparison, the turns are identified from the base dataset and the comparing dataset using Algorithm 1 . The identified turns are then compared with each other in the order of their occurrence. This comparison determines whether the user followed the instructions and took the advised turns or not. The last matching turn in both the datasets determined the minimum path covered by the user. The percentage of the path covered by the user is calculated based on when the last matching turn appears in the base dataset.

**Fig. 9:**
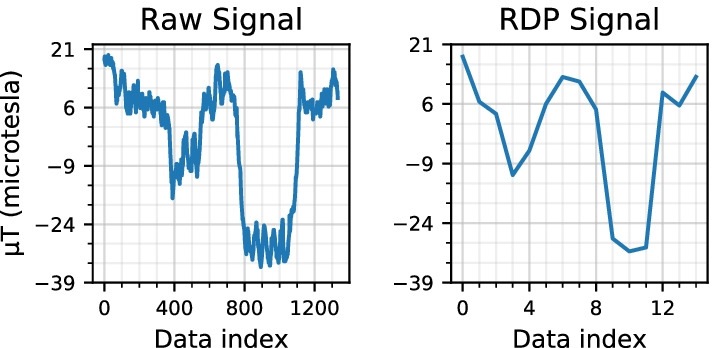
The plots show the conversion from the raw magnetic signal to the processed signal using the RDP technique to identify patterns

**Fig. 10:**
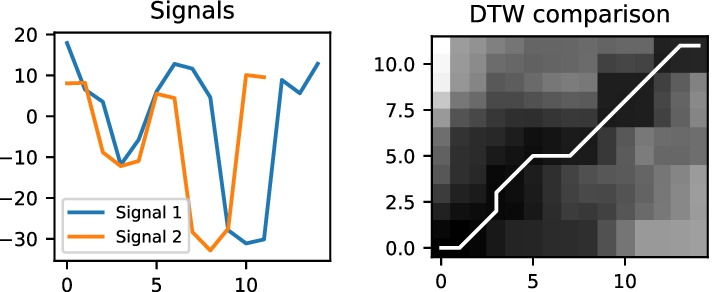
A comparison of two signals using the Dynamic Time Warping (DTW) technique to identify similarity. 45$^{\circ }$ lines show similarity in the two signals

1. *Magnetic data pattern matching* The magnetic data collected using the mobile application consists of magnetic latitude, magnetic longitude and magnetic altitude. Each of these signals has a different pattern based on the magnetic field in that direction. For example, if a user takes a right turn by moving from the west direction to the north direction, the latitude and longitude values are almost interchanged. However, the signal still contains magnetic field interference from the location. Thus in this work, the magnetic data is converted into a pattern that can detect local magnetic field changes. The *Ramer–Douglas–Peucker* (RDP) method is used with $epsilon = 5$ to identify the pattern in magnetic data. Figure 9 shows the conversion of magnetic latitude data into an RDP pattern. The patterns generated for two signals using the RDP method cannot be compared by simply overlapping them. Differences in the users’ walking speed can cause the pattern to occur at different times. Therefore, a Dynamic Time Warping (DTW) technique is used to compare the patterns to account for timing differences. An example of DTW matching is shown in Fig. 10. DTW measures the temporal similarity between two temporal sequences that may have a variance in speed. This technique produces a 2D matrix that stores the differences between each data value. Furthermore, a distance matrix is generated starting from an index of zero to the end of the matrix by adding the corresponding minimum difference value from the previous matrix. Finally, the minimum values in the distance matrix determine the similarity between the two datasets. The result from the distance matrix is further processed to calculate the percentage of the path covered.
2. *Wi-Fi Fingerprinting* Wi-Fi fingerprinting has been used extensively in indoor navigation systems that collect Wi-Fi information [39, 52, 54]. In this method, Wi-Fi’s and their corresponding RSSI values for different locations are compared, and a similarity score is determined. The location with the highest similarity score is determined to be the closest location. However, the number of Wi-Fi systems available and the differences in their RSSI values can significantly affect the score. For example, if a Wi-Fi is too far from the user, it may not always appear in the scan result and cause inaccurate estimation. Also, the RSSI values at a particular location may change due to obstructions in the sight line and cause significant differences in their RSSI values. To avoid this issue and reduce their impact on the estimation, the estimating process is split into two different techniques (1) Using all the filtered Wi-Fi information, explained in the next paragraph, and (2) Using the RSSI values of Wi-Fi’s with strong RSSI values among all locations, explained in the *Closest Wi-Fi location*. The latest Wi-Fi location information collected by the user is compared with each of the base dataset’s Wi-Fi locations. During the comparison, the percentage of matching BSSID is computed between the two locations. Occasionally, a distant Wi-Fi may appear in one or several locations along the path during a Wi-Fi scan. This unique Wi-Fi availability at particular locations can help in increasing the BSSID matching percentage. Also, the unique Wi-Fi availability can help shortlist the closest Wi-Fi location for a user. This similarity score is later used as a part of the final estimation to determine the user’s path.
  2 $$\begin{aligned} {\text {Loc}}_{\mathrm{i}} = [(bssid_{1}, rssid_{1}), \dots , (bssid_{n}, rssid_{n})] \end{aligned}$$
  3 $$\begin{aligned} {\text {LocDiff}}_{\mathrm{(i)}} = \sum _{k=1}^n \Big ( {\text {Loc}}_{\mathrm{base}}[bssid_{k}][rssid] - {\text {Loc}}_{\mathrm{i}}[bssid_{k}][rssid] \Big ) \end{aligned}$$
  4 $$\begin{aligned} {\text {Final Loc}} = {\text {MIN}} \Big ({\text {LocDiff}}_{\mathrm{(i)}}, \dots , {\text {LocDiff}}_{\mathrm{(n)}}\Big ) \end{aligned}$$
  5 $$\begin{aligned} {\text {LocDiff}}_{\mathrm{i}} = \sum _{k=1}^n \Bigg (Euclidean \Big ( {\text {Loc}}_{\mathrm{base}}[bssid_{k}][rssid], {\text {Loc}}_{\mathrm{i}}[bssid_{k}][rssid] \Big ) \Bigg ) \end{aligned}$$
3. *Closest Wi-Fi location* Researchers have proposed various approaches to identify the closest location using the BSSID and RSSI collected from different locations [38, 55, 59]. In this method, the BSSID or the media access control (MAC) address and the RSSI information of the access points are captured and stored along with the capturing location, Eq. (2). The user’s location is estimated by comparing the user captured Wi-Fi information with the pre-recorded database. Multiple approaches have been proposed in the literature to measure the difference between the two RSSI values of an access point [55, 59]. One of the common approaches is to calculate the sum of the differences between the RSSI values of an access point for different locations, Eq. (3). The location with the minimum difference is selected as the closest location, Eq. (4).

In this study, the data is first cleaned to remove the records with RSSI values larger than $-$30. This step is necessary to remove the noise and unrealistic values as explained in subsection *Data Cleaning*. The data is further filtered to select the Wi-Fi records with strong RSSI values and available in most locations. This filtration excludes Wi-Fis available in some locations and has weak RSSI values, as these cause large spikes in calculating the distance. The distance between the RSSI values is calculated using the Euclidean method. Thus, in the proposed system, the Eq. (3) is replaced with Eq. (5).

When selecting the closest Wi-Fi location, priority is given to the location with a minimum average distance. The second priority is given to the location with the maximum percentage of Wi-Fi matched.

**Fig. 11:**
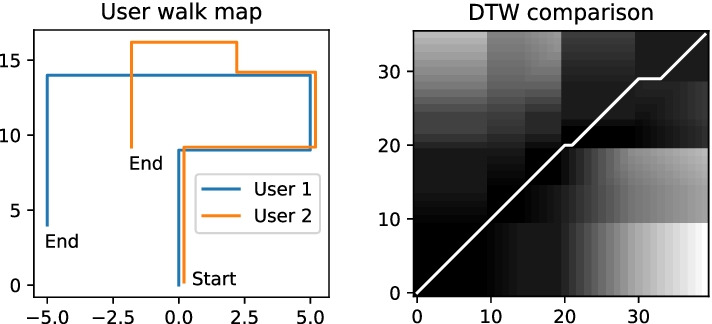
A comparison of the dynamic time warping (DTW) analysis between the two datasets

**Fig. 12:**
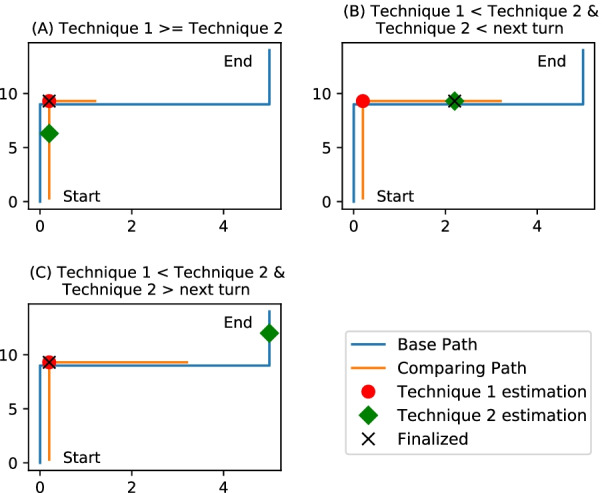
Result selection between *Technique 1 and 2*

#### Combining results from different techniques

Each of the four techniques discussed produces an estimation of the user’s location. These results are combined to predict the location of the user. The path covered from *Technique 2* is assumed to be the user’s location. In *Technique 2*, the DTW method matches data patterns in the processed magnetic data to find the overlapping patterns with the user’s data. However, this technique can over-match the data, which leads to inaccurate estimations. For example, Fig. 11 shows the path walked by two users. Both users started at the exact location and followed the same path, however, in the middle of the track *User 2* diverged from the path of *User 1* and ended up in a different location. The DTW analysis between the two user tracks shows that the users walked the same track with slight deviations. This issue of over-matching is managed by considering the results of *Technique 1*.

*Technique 1* compares the turns identified in the dataset in the order of their occurrence. This comparison identifies the differences between the two datasets and returns a percentage of the path that matches. The result from this technique confirms that the user took turns similar to the comparison dataset. The user location is estimated based on the following rules: Rule 1, if the result of *Technique 1* is larger or equal to the result of *Technique 2*, then consider the result of *Technique 1*, as the turn detection method is more accurate then the DTW matching, shown in Fig. 12A. Rule 2, if the user’s path covered from *Technique 1* is less than the user’s path covered from *Technique 2*, it means that the DTW matching predicts the user has walked ahead of the last turn. It might be possible that the missed turn is due to DTW over-matching. To confirm that it is not over-matching, the user’s path covered from *Technique 2* should be before the next turn’s location. If the previous statement is true, then the user’s location is estimated to be the user’s path covered from the turn detection method (*Technique 1*), otherwise user’s path covered from the DTW matching method (*Technique 2*), shown in Fig. 12B, C.

The results from *Techniques 3 and 4* provide the closest Wi-Fi location to a user. These results help achieve higher accuracy in locating the user, especially when they are walking on a straight path with no turns. For comparing the results of these techniques, a set of possible Wi-Fi locations are identified from the base dataset. These locations include all the Wi-Fi locations between the previous turn to the next turn. If the result from *Technique 3 and 4* matches within the list of possible Wi-Fi locations, then the matching Wi-Fi location is estimated to be the user’s location. If no match is found from the list, it confirms that the user is not on track.

#### Testing environment and strategy

The testing for the proposed system was performed in a home with two floors. This home was selected primarily due to COVID-19 restrictions that limit access to office buildings and malls. This home is also located in a neighbourhood with many Wi-Fi connections available at a variety of distances. The availability of many Wi-Fi connections creates a similar environment to that of an office building or mall. With the two floors in the home, multi-floor navigation can also be tested. Some small start-up companies and community offices in remote locations use homes as their office space. They also store AEDs for public use, by selecting a home for experimentation we are simulating a similar environment.

The travel path selected for experimentation is shown in Figure 3. The path was selected to incorporate floor changes, long straight paths, different turns in the pathway, walking next to electrical and magnetic devices, and covering the maximum distance without overlapping. In the planned path, the user has to walk 7.3 m straight, take a right turn, walk straight for 5.4 m, take a left turn to climb upstairs, and have a U-turn. Upon reaching the first floor, the user takes another U-turn and walks 4.2 m straight to reach the AED location.

Three participants used an Android mobile phone having the android application shown in Fig. 2 to collect the data. Each participant walked the path 20 times to collect the data.

A cross-comparison among the datasets was performed, each dataset collected was compared against all the other datasets. This type of analysis is performed to compare datasets collected by the same user and with other participants. The comparison also includes partial datasets to verify the user’s location on the path.

#### Computational resources

During experimentation, two devices were used to capture the information and process the results. First, an Android mobile (Google Nexus 5X with 2 GB RAM and Android 8.1.0 installed) for capturing the magnetic and Wi-Fi information during a user’s walk. Second, a laptop with an Intel i7 processor (8 core), 40 GB memory, and 1TB Solid State Drive to compare the datasets. The laptop will eventually be replaced with a cloud-based server to perform the analysis.

## Results

**Fig. 13:**
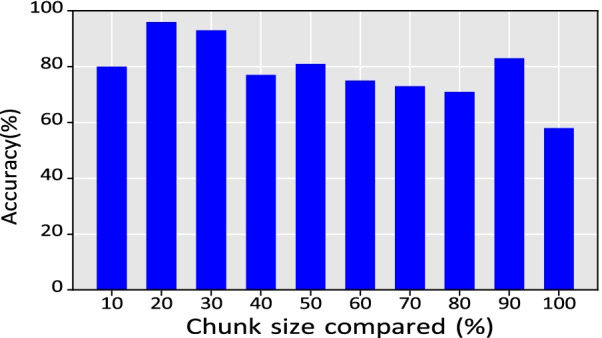
The graph shows the accuracy achieved by the proposed system when segments of datasets were compared

**Fig. 14:**
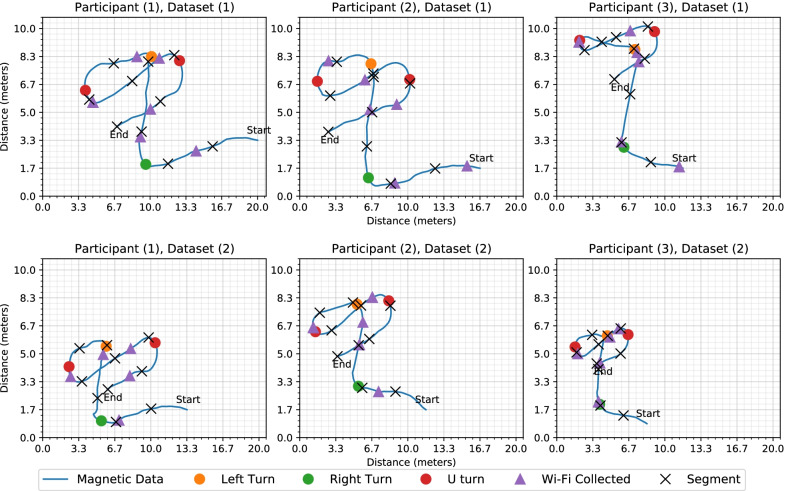
The subplots show the map generated for all 3 users and 2 of their datasets using the magnetic information. The maps are marked with the turns detected by the system and the positions on the path where Wi-Fi information was collected

The experimentation dataset contains data from three participants who walked 20 times on the path shown in Fig. 3. A total of 60 datasets were collected. A cross-comparison of datasets is performed to measure the accuracy between intra-user datasets. However, this analysis compares the entire dataset and misses the potential partial matching of the dataset for identifying a user’s location on the path. Therefore, each dataset is segmented into ten parts from 10 to 100% with a step of 10% (i.e., 0–10%, 0–20%, ..., 0–100%). A total of 36,000 comparisons were performed (i.e., 60 datasets were cross-compared, each with 10 segments, $60\times 60\times 10=36{,}000$).

Combining results from different techniques, the proposed system achieved an accuracy of 79%, determining the path covered by a user with an error distance of ± 2.4 m. Figure 13, shows the accuracy achieved by *AEDNav* for the different segments of data. The accuracy was lowest (i.e., 58%) when the full segment was compared with the base dataset.

Another reduction in the *AEDNav* accuracy occurs during segments 60%, 70%, and 80%. The lower accuracy is again observed during a straight line walk when only the Wi-Fi was accessed once along this path. As described earlier, due to a lack of data reference points along the path, the system is unable to estimate the user’s location precisely. The accuracy improved when the 90% segment was compared. This increase in the accuracy is due to an increase in the number of Wi-Fi scans completed and a turn is detected.

**Fig. 15:**
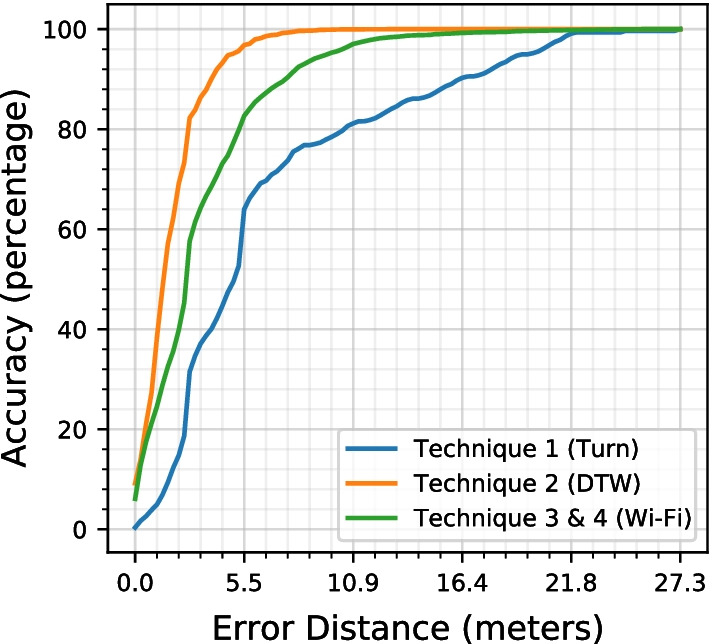
The graph shows the accuracy achieved by the various techniques

Figure 15 shows the accuracy achieved by the individual techniques proposed in this study. *Technique 2* provides the highest accuracy among all of the techniques. However, this technique’s results are not reliable, as previously discussed in the *Methods* section. *Technique 3 and 4* provided the next best accuracy followed by *Technique 1* Turn detection. *Technique 1* was able to achieve an accuracy of 80% with an error distance of ± 9.4 m, *Technique 2* with error distance of ± 2.4 m and *Technique 3 and 4 * with error distance of ± 4.6 m.

Figure 14 shows the maps generated using the magnetic information, along with the Wi-Fi scan locations of six different datasets collected during this research. Two datasets for each participant are plotted to demonstrate the variations in their walks. All users travelled the same marked path at their average walking speed. If a user’s speed is consistent and they travel a path twice, then the plot for their walks should be overlapping. However, in Fig. 14, it can be observed that there is a considerable difference between the plots within and among users. These plots are created assuming all the users are walking at a consistent speed. In the map for Participant 1, the distance between the Right turn and the Left turn is significant (approximately 1.8 m). The reason for the difference in distance is the variation in the walking speed of the user. However, the distance between the two U-turns for the two datasets for Participant 1 is similar (7.3 and 7.9 m). There was slight variation in the user’s walking speed between these sections. Since there can be considerable differences in a user’s walking speed, the *AEDNav* uses the path travelled patterns to estimate the user’s location rather than calculating the distance.

**Fig. 16:**
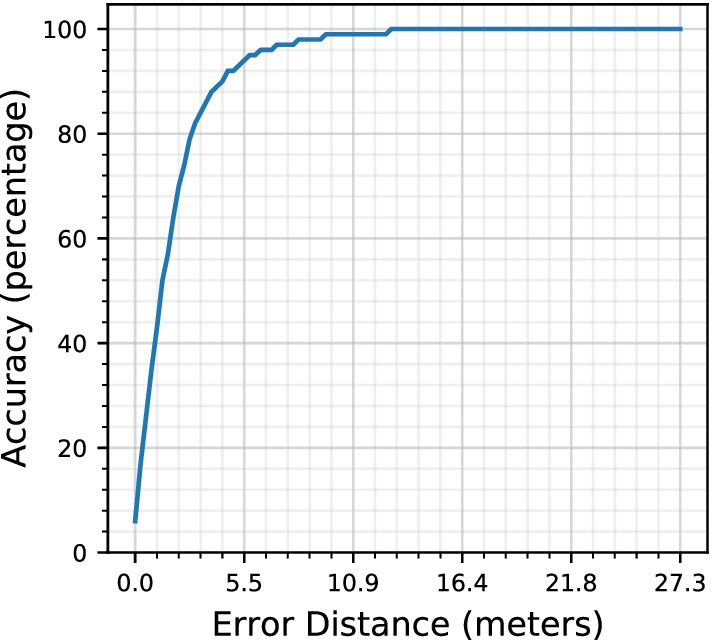
The graph shows the relationship between system accuracy over a range of error distances

Figure 16 shows the accuracy achieved by *AEDNav* over the different error distances. When the error distance is set to zero, the system can achieve an accuracy of 6% in estimating the user’s location. The error distance of ± 2.4 m allows the system to reach an accuracy of 79%. Increasing the error distance increases the accuracy, however, the increase in error will affect the navigation instructions for the user and thus reduces the applicability of the system for indoor navigation.

**Table 2:** A comparison between the proposed system and existing systems

	AEDNav (Proposed system)	You Li (2015)
Accuracy	86%	80%
Error distance (m)	4	4
Data collected	A walk from start to the end of path	Half an hour of walk on the path

For the comparison of system accuracy, the one developed by Li et al. [54] is the most similar to *AEDNav*. Both systems use Wi-Fi and magnetic information to provide indoor navigation [60]. The system proposed by Li et al. achieved an accuracy of 80% with an error distance of ± 4 m, and the *AEDNav* achieved an accuracy of 86% with the same error distance. Table 2, shows the comparison between *AEDNav* (the proposed system) and Li et al.’s proposed system. A significant difference between the two systems is that *AEDNav* uses data from a single walk to provide navigation. In contrast, Li et al.’s system required 30 min of walking to develop the baseline map to provide navigation.

## Discussion

During a sudden cardiac arrest event, it is crucial to provide early resuscitation to the patient by performing CPR and applying an AED. The reduced survival rate of 10% per minute necessitates quick action from bystanders. Therefore, it is vital to provide resuscitation in the minimum possible time. Emergency service providers and communities understand the importance of early resuscitation and have implemented multiple solutions to ensure quick treatment times. Some of the solutions are: implementing responder network systems, increasing the availability of AEDs in public places, and mobile applications to provide the location of nearby AEDs.

The selection of the nearest AED can be performed either by the user manually or by using the responder network system that has been developed earlier by the authors [61]. By providing only the AED location, the user may have to spend significant time finding the AED within the premises. There are many existing indoor navigation solutions available in the literature. However, they are not feasible for an extensive network of AEDs within a city because they either require enormous data or special hardware on the premises. The authors could find only one proposed system that uses Wi-Fi and magnetic data to provide indoor navigation in the literature [60]. A primary reason for the limited use of these technologies is that the data often contains significant noise that can make the data unusable. Another reason is that these technologies independently cannot accurately provide the localization of the user.

This research proposes an indoor navigation system called *AEDNav* that does not require special hardware to be installed on the premises, nor requires a large quantity of data to be collected. The *AEDNav* requires a user to walk from the building entrance to the AED with their smartphone running the data collection application. The application captures the information about the existing Wi-Fi devices and magnetic information of the path and transmits it to the server to build the map. This walk can be performed by the AED installer or the AED owner, as they are the first person to know the location of the AED.

The accuracy of the system is adequate to provide navigation within the building. When a user is walking in a straight line, the system’s accuracy with an error distance of ± 2.4 m is not a concern. However, estimates are crucial when a user is near a turn or about to reach their destination. In both cases, the error distance of ± 2.4 m can be corrected by the user upon examining the environment. For example, if a user is a few meters behind or ahead of the turn, they can examine the environment and decide to take the nearest turn. Also, the corridors in a building are typically 1.8 m wide on average. Thus, the significance of the error distance is further reduced. Another issue with accuracy occurs when the user reaches the end of their path. This problem is minimized because the AEDs are labelled to ensure they can be identified from a distance. Thus, a user can evaluate the environment and find the AED from a distance.

There are several reasons for the low accuracy that we observed in our experiment. First, the DTW comparison performs pattern matching and ignores the length of data in each pattern. The walk between segments 90% and 100% of the path is a straight line walk, and thus the DTW matching fails to estimate the user’s location. Second, the distance between segments 90% and 100% of the path was covered in less than 4 s by the user. Four seconds was insufficient time for the smartphone to scan the Wi-Fi access points multiple times. With only information from a single Wi-Fi location, the Wi-Fi techniques could not be used to achieve higher accuracy. The accuracy at the end of the path can be ignored because the AEDs are easily identified from a distance. Furthermore, the user would likely be within ± 2.4 m from the AED.

Due to the COVID-19 restrictions, the *AEDNav* system was tested in a home environment with all possible conditions that mimic real world scenarios, such as stairs, large electrical appliances and multiple Wi-Fi signals. When the restrictions are relaxed, the authors intent to test the system in different physical locations to further strengthen the case for real time implementation. The system was tested using the data collected from a single walk of the path. In theory, if the data of multiple paths are combined, then the accuracy in locating a user will further increase. In the future, the system will be further developed to include other technologies that may be available on the premises, such as beacons or RFIDs. The information from these devices can provide more reference points along the path. A step counter can overcome the existing limitation of estimating a user’s location on a straight-line path may also be beneficial to the system.

The *AEDNav* was developed to provide indoor navigation to an AED. However, this system can also be used for other purposes to provide indoor navigation (e.g., conferences, malls, and offices). Another critical implementation of this system is for the fire department. This system could potentially be used by emergency services such as firefighters or paramedics to track their colleagues while in an unknown environment to ensure their safety. The *AEDNav* system can serve both as mapping as well as tracking system within an indoor environment.

## Conclusion

This research aimed to develop the *AEDNav* system to provide indoor navigation to an AED. The *AEDNav* uses Wi-Fi and magnetic information to build a path that can then provide navigation instructions to a user. The proposed system uses various techniques to localize the user along the path. The novelty of the *AEDNav* system requires the AED installer/owner to walk the path a single time to provide the baseline navigation route, and no special hardware is required. Reducing the amount of data required to map the initial route makes it feasible for an individual to record the data and reduce overall computation time. The *AEDNav* outperforms some of the existing systems that use similar technologies but require larger datasets.

## Data Availability

The datasets used and analyzed during the current study are available at https://github.com/rao-g/AedNavDataSource.

## Abbreviations

SCA: Sudden cardiac arrest
CPR: Cardiopulmonary resuscitation
AED: Automated external defibrillator
RNS: Responder network system
RFID: Radio frequency identification device
QR code: Quick response code
AI: Artificial intelligence
MEMS: Micro electro mechanical systems
RSSI: Received signal strength indicator
GPS: Global positioning system
RDP: Ramer–Douglas–Peucker
DTW: Dynamic time warping
BSSID: Basic service set identifiers
